# Role and Correlation of High Resolution Ultrasound and Magnetic Resonance Imaging in Evaluation of Patients with Shoulder Pain

**DOI:** 10.12659/PJR.901540

**Published:** 2017-07-28

**Authors:** Amandeep Singh, Chuni Lal Thukral, Kamlesh Gupta, Mahesh Inder Singh, Sneh Lata, Ram Krishan Arora

**Affiliations:** 1 Departmen to Radiodiagnosis and Imaging, Shri Guru Ram Das (SGRD) Institute of Medical Sciences and Research, Amritsar, Punjab, India; 2 Departmen to Orthopaedics, Shri Guru Ram Das (SGRD) Institute of Medical Sciences and Research, Amritsar, Punjab, India

**Keywords:** Magnetic Resonance Imaging, Rotator Cuff, Shoulder Joint, Tears, Ultrasonography

## Abstract

**Background:**

The study aimed to evaluate of the role of high-resolution ultrasound and magnetic resonance imaging in patients with shoulder pain.

**Material/Methods:**

This prospective study included 50 patients referred for ultrasound and MRI because of shoulder pain. All patients were examined clinically, followed by radiography of the affected shoulder. High-resolution ultrasound examination of the involved shoulder was performed together with an examination of the contralateral normal shoulder, followed by MRI of the symptomatic shoulder in all 50 patients.

**Results:**

In the present study, the majority of patients were in age group 56–65 years, 56% were males and 44% were females (of a total of 50 patients). A total of 40 patients were diagnosed as having rotator cuff tears on ultrasound (USG) and MRI. USG showed complete-thickness tears in 25 patients and partial-thickness tears in 15 patients. MRI detected 28 complete- and 12 partial-thickness tears of the rotator cuff. In the diagnosis of rotator cuff tears, the strength of agreement between ultrasound and magnetic resonance imaging was good (kappa coefficient=0.79).

**Conclusions:**

Ultrasonography of the shoulder shows promising results in the diagnosis of rotator cuff tears and in differentiating partial from complete tears. A wide availability, cost effectiveness and better tolerability of ultrasonography make it a modality of first choice for evaluating rotator cuff tears.

## Background

Rotator cuff pathologies, particularly rotator cuff tears, are a common cause of pain involving the shoulder. Clinical examination alone has a limited value in deciding on the management options for the underlying aetiology. The decision on either conservative management or surgical treatment depends on an accurate diagnosis and severity of any underlying tear of the rotator cuff [1,2].

The preferred imaging modalities for evaluation of suspected tears of the rotator cuff include high-resolution ultrasound (USG) and magnetic resonance imaging (MRI). Both these modalities have their own merits and demerits. Accuracy, availability, cost effectiveness and expertise are some of the important parameters that guide the process of making a decision on the best modality [3].

There have been studies done in the past that evaluated the accuracy of either ultrasound or MRI in detection of tears of the rotator cuff and only few studies compared these two methods.

Low cost, wide availability and scan dynamics are some of the advantages in favour of shoulder ultrasonography. A review of relevant literature shows variability in the accuracy of ultrasonography in differentiation between complete- and partial-thickness tears [4–7].

MRI has a multiplanar capability providing great soft tissue information [8]. It is proven to have both high sensitivity and specificity for the evaluation of tears of the rotator cuff [8,9].

### Aims and objectives

Evaluation of role of high-resolution ultrasonography and magnetic resonance imaging in patients with shoulder pain.

## Material and Methods

This was a prospective study that included fifty patients who were referred to a radiology department for high-resolution ultrasonography and magnetic resonance imaging because of shoulder pain. All patients were examined clinically, followed by radiography of the affected shoulder. High-resolution, real-time ultrasound examination of the involved shoulder was done together with an ultrasound examination of the contralateral normal shoulder for comparison in all fifty patients. All patients underwent magnetic resonance imaging of the symptomatic shoulder.

The following patients were excluded from the study:

1. Those with contraindications to MRI
2. Patients with known or diagnosed fracture/dislocation involving the shoulder on plain radiography.
3. Patients who had undergone shoulder surgery for any reason

### Study duration

The study was performed from September 2013 to December 2015.

### Ultrasonography technique and patient position

#### Technique and equipment

Ultrasound examinations were performed with the VOLUSON E8 EXPERT BT09(GE), equipped with a SP10-16-D wide-band linear transducer with a frequency of 7–18 MHz.

#### Patient position

The position of the patient for ultrasonography of the shoulder differs among institutions, countries and examiners.

In this study, the patient was asked to sit on a revolving stool with the examiner seated opposite on a similar stool. The height of the patient’s stool was adjusted to be ergonomically comfortable for scan performance.

### Long head of biceps tendon

Patient’s forearm was placed in a slight internal rotation with the palm of hand facing upwards and elbow flexed at 90 degrees. The bicipital groove was identified. The biceps tendon was seen between the tuberosites, i.e. the greater and lesser tubersosity. Scanning was done in short and long axes planes and the biceps tendon was followed from its intraarticular course down to the muscle belly [10]. Normal tendon was seen as a uniform fibrillary structure.

### Subscapularis (SSC) tendon

The arm was rested in a position with the elbow fixed on the iliac crest on the same side. With the palm of hand faced upwards, the probe was placed in a transverse plane at the bicipital groove and the arm of the patient was externally rotated. This tendon was examined in transverse planes and sagittal planes with passive internal and external rotation. The SSC tendon is visible when entering medially to the groove [10]. It was seen as an elongated and slightly convex tendon.

### Supraspinatus tendon

The dorsal surface of the hand was placed on the back pocket of the opposite side with elbow opposed to the lateral wall of the chest. This position made the supraspinatus tendon project anteriorly. Scanning was done in both transverse and longitudinal axes. The subacromial-subdeltoid bursa was seen in-between the deltoid and supraspinatus as a thin hypoechoic structure [10].

### Infraspinatus tendon

The palmar surface of the hand was placed on the opposite shoulder with the ultrasound probe placed over the posterior aspect of the glenohumeral joint. Supraspinous and infraspinous fossae were identified with upward and downward movement of the probe using the scapula spine as a landmark. The infraspinatus muscle was seen separately from the teres minor muscle within the infraspinous fossa.

### MR technique and protocol

MR scan was carried out on a Philips Gyroscan, Achieva 1.5 Tesla unit. The standard imaging protocol consisted of axial T1W coronal, T2W FFE sagittal, T2W SPAIR coronal, PD SPAIR coronal, PD SPAIR sagittal and STIR coronal sequences (Table 1).

### Image analysis

The images were analysed by a senior radiologist with 5 years of experience in interpreting USG and MRI scans of the shoulder. MRI was done after USG findings were noted.

### The following classification of rotator cuff tears was applied

Classification of tear:

- No tear seen.
- Partial-thickness
  – Articular side tear.
  – Bursal side tear.
- Full-thickness tear
  – Without retraction.
  – With retraction.

Ultrasound findings in full-thickness tears:

1. Lack of visualization of the tendon.
2. Complete discontinuity or breech (seen as focal hypoechoic defect or a mixed echogenicity defect which extends through the entire thickness of the tendon)
3. Focal thinning or loss of some substance of the tendon and appreciable irregular margins of tear.

Ultrasound diagnosis of partial-thickness tear:

1. Focal discontinuity which involves partial thickness of the tendon (seen as focal hypoechoic defect or a mixed echogenicity defect which extends through the part of thickness of the tendon) on either the articular or bursal side or within the tendon.
2. Focal thinning of the tendon or loss of the normal convex contour on the outer border (bursal surface) of the tendon.

Findings of MRI were classified on the basis of:

1. Full-thickness tear (with/without retraction).
2. Partial-thickness tear (articular or bursal side).

## Results

The study group with a clinical suspicion of rotator cuff tears comprised of a total of 50 patients with age ranging from 15 to 65 years. The majority of patients belonged to the age group between 56 and 65 years, comprising 36% of all patients (Table 2). Men (56%) outnumbered women (44%) and the right shoulder was involved in the majority of patients (77%).

On ultrasound, of 50 patients, 25 (50%) patients had full-thickness tears, 15 (30%) partial-thickness tears, 9 (18%) patients had tendinosis and 1 (2%) patient did not have any abnormality. Out of the 15 patients with partial-thickness tears, 9 patients showed hypoechoic defects that involved the articular surface and 6 patients showed hypoechoic defects that involved the bursal surface. Out of 25 patients with full-thickness tears, 13 patients had tears without retraction and 12 patients had tears with retraction (Table 3).

On MRI conducted in 50 patients, 12 (24%) patients had full-thickness tears, while 28 (56%) patients had partial-thickness tears, and 10 (20%) patients had tendinosis. Thus, in 40 patients MRI showed rotator cuff tears. In 12 patients diagnosed with partial-thickness tears, 7 patients showed defects involving the articular surface and 5 patients had defects along the bursal surface. In 28 patients diagnosed with full-thickness tears, 13 patients had full-thickness tears without retraction and 14 patients had full-thickness tears with retraction (Table 3)

Of 50 patients, in 4 (8%) patients the biceps tendon in the intertubercle sulcus was not visualised on usg and the same results were seen on MRI. In 13 out of 50 patients, biceps tendon effusion was seen in 6 patients (46%) on USG and in 7 patients (54%) on MRI.

The kappa coefficient was used to assess the agreement between USG and MRI. The strength of agreement on rotator cuff tear diagnosis is considered as good (k=.79)

The most common finding associated with tears of the rotator cuff was tendinitis. Other associated findings include acromio-clavicular joint degeneration and glenoid labral tears (Table 4).

## Discussion

Rotator cuff tears are a frequent finding in patients with shoulder pain. Non-invasive imaging modalities such as ultrasonography and MRI are used for evaluating rotator cuff pathologies. USG can be used as a primary modality because its accuracy in detecting partial- and full-thickness rotator cuff tears is comparable to MRI, as mentioned in literature. Our data were analysed with the kappa values.

Out of 50 patients, 28 were males (56%) and 22 (44%) were females Thus, the M: F ratio was 1.3: 1. Our study found no statically significant differences in the prevalence of rotator cuff tears between genders, which correlates with the results of the study carried by Milgrom et al. [11].

In the present study, 35 patients (70%) had pain involving the right shoulder and 15 patients (30%) had pain involving the left shoulder. Thus, the right shoulder was more frequently involved than the left shoulder. This correlates with the results of the study by Bouaziz et al. [12] who found right shoulder involvement (68%) to be more frequent than left shoulder involvement (32%).

**Figure 1:**
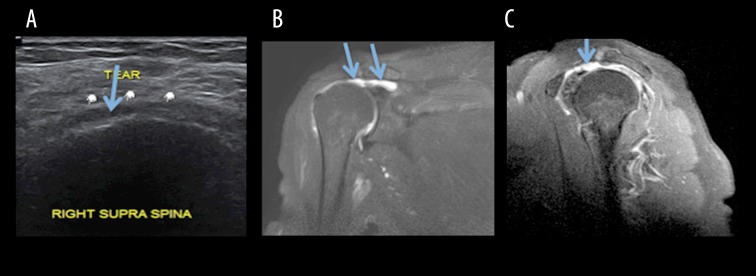
(**A**) USG image showing a complete tear of the supraspinatus tendon (**B**) MRI, T2W coronal image (**C**) T2W, sagittal image depicting a full-thickness tear of the supraspinatus tendon (arrow marked).

**Figure 2:**
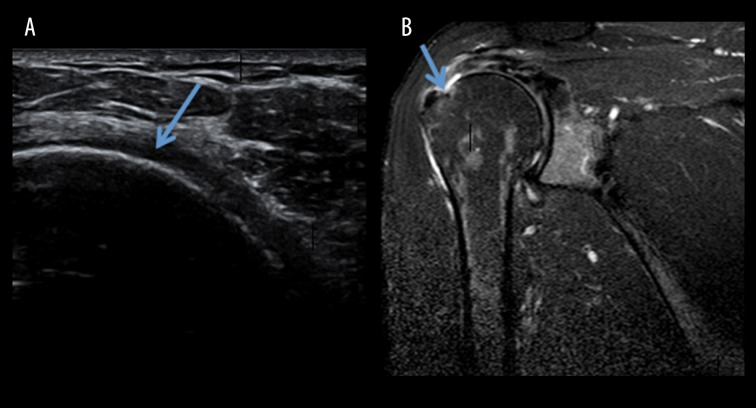
(**A**) USG image depicting a partial-thickness tear of the supraspinatus tendon (**B**) MRI, T2W coronal image depicting a partial-tear of the supraspinatus tendon (arrow marked).

The tears of the rotator cuff, as seen in this study, were commonly visualised as hypoechoic, full-thickness defects in the tendon extending from the bursal surface to its articular margin (Figures 1, 2). Partial-thickness tears on USG appear as focal discontinuities at the articular or bursal aspect or are located within the substance of the tendon. On MRI, full-thickness tears are seen as hyperintense defects in tendons on T1W images. The presence of hyperintense fluid at the site of tears on T2-weighted images supports the diagnosis (Figures 1, 2). Additional signs of complete tears include muscle retraction, fluid in the subacromial-subdeltoid bursa and displacement of the peribursal fat plane.

**Figure 3:**
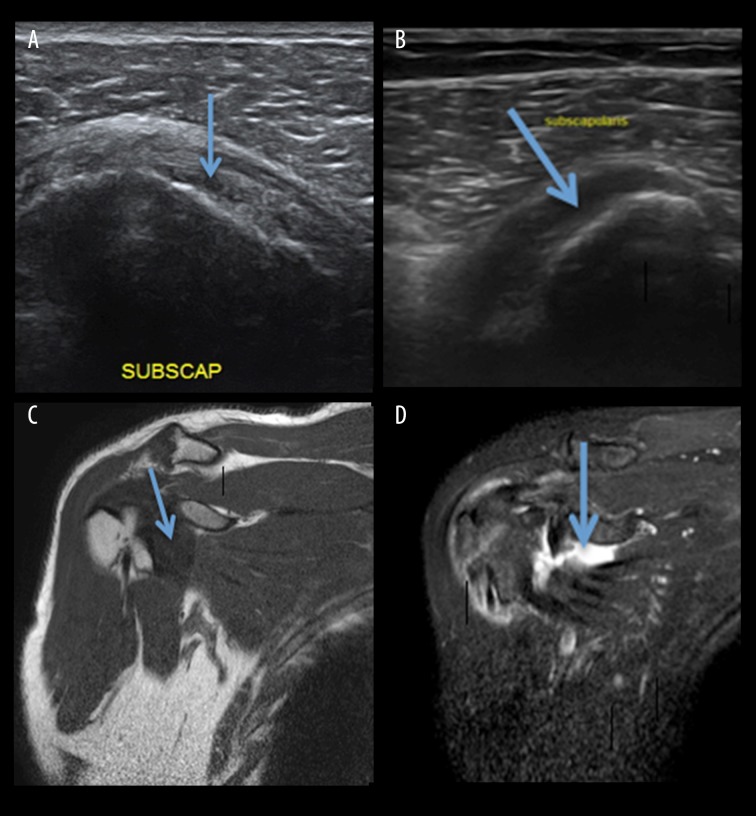
(**A**) USG image showing a normal subscapularis tendon (**B**) USG image showing a complete tear of the subscapularis tendon (**C**) MRI, T1W coronal image depicting a normal subscapularis tendon (**D**) MRI, T2W coronal image depicting a full-thickness tear of the subscapularis tendon (arrow marked).

An ultrasound examination revealed rotator cuff tears in 40 patients. Similar results were reported by Brandt et al. [13]. Singisetti et al. [14] found that, in detection of supraspinatus tendon tears, ultrasonography attained sensitivity of 89% and specificity of 43%. For detection of subscapularis tendon tears (Figure 3), ultrasonography attained sensitivity of 30%. Sensitivity, specificity and predictive values were good in larger full-thickness tears but were significantly reduced in subcentimetre and partial-thickness tears, particularly those involving the tendon of the subscapularis muscle.

In the prospective study by Kamath et al. [15], sensitivity, specificity, positive predictive value and negative predictive value for the diagnosis of rotator cuff tears using ultrasound was 70.6%, 90.6%, 85.4% and 76.35% for supraspinatus, infraspinatus, teres minor and subscapularis tendon injuries, respectively. The authors concluded that ultrasound is a non-invasive method to confirm a clinical diagnosis.

In the present study, MRI showed rotator cuff tears in 40 patients of whom 12 patients had partial-thickness tears and 28 patients had full-thickness tears. Similar results were reported by McMonagle et al. [16], Fischer et al. [17] showed that ultrasonography and MRI are comparable and ultrasound is beneficial in revision cases. In their study, accuracy of 91.1% was seen for detection of tears of the supraspinatus tendon, 84.4% for the infraspinatus tendon and 77.8% for the subscapularis tendon.

**Figure 4:**
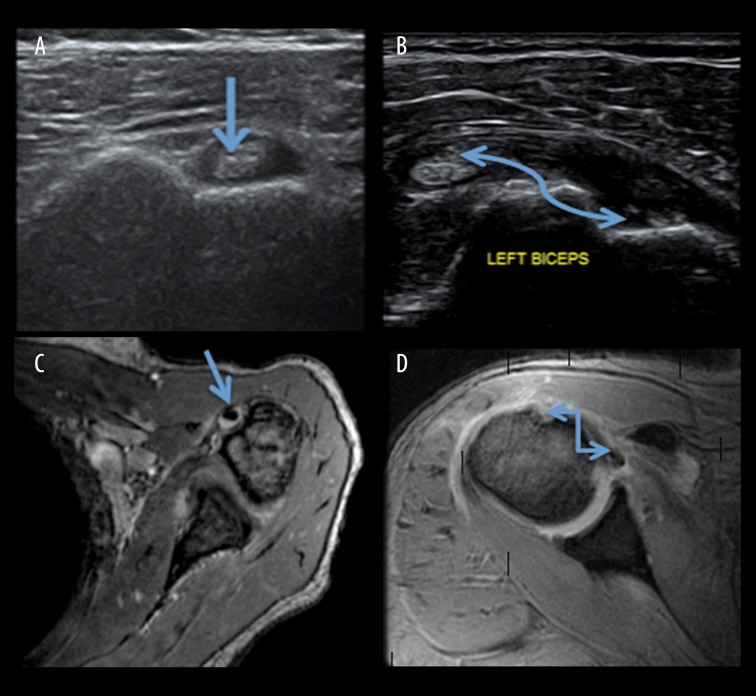
(**A**) USG image showing a normal biceps tendon in the bicipital groove (**B**) USG image showing non-visualization of the biceps tendon in the bicipital grove (**C**) MRI, T1W axial image depicting a normal biceps tendon in the bicipital groove (**D**) MRI, T2W axial image depicting non-visualization of the biceps tendon in the bicipital grove (arrow marked).

**Figure 5:**
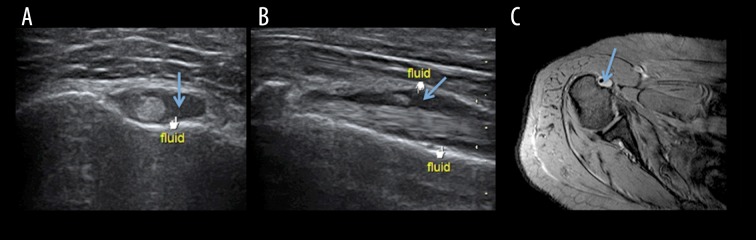
(**A**) USG, axial image showing a mild fluid accumulation in the bicipital groove (**B**) USG, longitudinal image showing a mild fluid accumulation along the biceps tendon (**C**) MRI, T2W axial image depicting a mild fluid accumulation in the bicipital groove.

In the present study, in 6 (12%) cases bicep tendon sheath effusion was seen on usg examination, while on MRI 7 (14%) cases of bicep tendon sheath effusion were seen (Figures 4, 5). Thus, USG and MRI showed a high agreement for detection of bicep tendon sheath effusion. Similar results were reported by Alasaarela et al. [18] who reported effusion of bicep tendon sheath in 24 shoulders on MRI and in 20 patients on USG. They found a good agreement between USG and MRI for detection of effusion of the biceps tendon sheath. Chen H et al. [19] examined 125 patients to compare physical examination with musculoskeletal ultrasound in diagnosing long head tendinitis and concluded that these tests were limited by poor sensitivity. US can be an image modality of choice in diagnosing biceps pathology.

Non-visualization of the biceps tendon in the bicipital grove indicates either rupture or subluxation of the tendon (Figure 4). In the present study, 4 (8%) patients had non-visualized biceps tendons in the intertubercle sulcus on usg and the same results were observed on MRI. In a study by Walch et al. [20], 16% of cases showed a medial displacement of the biceps tendon (long head).

Bhatnagar et al. [21], in their study on musculotendinous pathologies of the shoulder joint, found that MRI was successful in an overall assessment of the joint structure. Its ability to evaluate the labrum and various glenohumeral ligaments cannot be superseded by USG.

Out of 50 patients, 40 patients were diagnosed with rotator cuff tears of whom 25 patients had full-thickness tears and 15 patients had partial-thickness tears on USG. When MRI was conducted in these patients, it showed 28 full-thickness tears and 12 partial-thickness tears, which means that 3 patients were falsely diagnosed with partial-thickness tears on USG that turned out to be full-thickness tears on MRI. Out of the remaining 10 patients, 9 patients were diagnosed with tendinosis (1 patient did not have this finding) on USG but MRI showed tendinosis in all 10 patients. This means that 1 patient was falsely diagnosed by means of USG. Our study assessed the agreement between the two methods using the kappa coefficient. There was a good agreement (k=.79) between USG and MRI. Similar results were obtained by Alasaarela et al. [18].

According to Saraya et al. [22], ultrasound was as accurate as MRI for assessment of tears of the rotator cuff, both full- or partial-thickness tears. They also concluded that due to its lower cost, it may be a useful imaging modality which is cost-effective, provided the examiner has adequate expertise or training.

Roy et al. [23], in their meta-analysis, confirmed a similar and high diagnostic accuracy of ultrasonography, MRI and MR arthrography in the characterisation of full-thickness rotator cuff tears in individuals with shoulder pain.

## Conclusions

Based on our results, it can be concluded that ultrasonography is an effective imaging modality that has a positive effect on the management of many patients presenting with shoulder pain and/or disability. Shoulder ultrasound has high accuracy in diagnosing tears of the rotator cuff and in differentiating partial- from full-thickness tears. There was a good agreement (kappa value=0.79) between USG and MRI in diagnosing rotator cuff pathologies. A wide availability, lower cost and better tolerability of ultrasonography make it a modality of first choice for evaluation of rotator cuff tears. MRI can be reserved for patients with suspicious USG results.

## Figures and Tables

**Table 1:** MRI sequences.

SEQUENCES	TR	TE	THK	FOV	RFOV	NSA
STIR COR	4927	60	3.0/1.0	160	100%	3
T1W TSE COR	500	20	3.0/0.3	170	100%	3
PD SPAIR COR	3027	20	3.0/0.3	160	91%	2
T2W 3D FFE TRA	36	18	2.0/1.0	180	90%	1
PD SPAIR SAG	3020	20	3.0/0.3	160	90%	2
T2W TSE SPAIR COR	2280	60	3.0/0.3	170	100%	4

**Table 2:** Age incidence.

Sr.no	Agegroup (in years)	Number of patients	Percentage
1	<25	3	6
2	26–35	5	10
3	36–45	9	18
4	46–55	15	30
5	56–65	18	36
6	**Total**	**50**	**100**

**Table 3:** Rotator cuff tears (n=40) on ultrasound (USG) and MRI.

Tendon involved	Partial tear		Complete tear	
	USG	MRI	USG	MRI
Subscapularis	3	1	5	4
Supraspinatus	12	11	18	21
Infraspinatus	0	0	2	2
Teres minor	0	0	0	0
**Total**	**15**	**12**	**25**	**27**

**Table 4:** Associated findings in patients with rotator cuff tears.

Associated findings	No. of patients
Tendinitis	18
Glenoid labral injuries	8
Acromio-clavicular joint degeneration	5
